# Windows of opportunity: a new tree-shrub dynamic at alpine treeline?

**DOI:** 10.1093/nsr/nwad212

**Published:** 2023-07-29

**Authors:** Grant P Elliott

**Affiliations:** Department of Geography, University of Missouri, USA

Of increasing importance while the climate is under the influence of chronic environmental change driven by rising levels of CO_2_, temperature and vapor pressure deficit (VPD), is understanding how vegetation will respond over space and time as we cross critical thresholds [1]. This is especially true in mountain regions where amplified rates of warming are expected to affect the elevational zonation of plant communities [2]. A recent meta-analysis (n = 143) found alpine treeline, the uppermost extent of the mountain forest belt along the boundary with alpine tundra, has advanced upslope at most sites across the Northern Hemisphere (88.8%) since 1901, yet at a relatively slow rate of 0.354 m/yr [3]. This rate is approximately half of what is expected from rising temperature influences alone [3]. At a hemispheric scale, rates of treeline advance were best explained by autumn precipitation and winter warming [3]. Shrubs commonly exist beyond the treeline, and widespread shrubline advance was reported across arctic and alpine tundra in response to climate warming [4]. This is notable because the presence of shrubs can strongly influence tree establishment at the treeline through facilitation and/or competition, depending on shrub density and local filters of climate including slope aspect [5,6]. Together, this highlights the necessity of elucidating tree-shrub dynamics as the climate continues to warm, particularly for predicting future forest cover since trees sequester more carbon than shrubs.

Li *et al.* [7] address this with long-term cambial growth phenology of sympatric trees and shrubs at two Tibetan Plateau treeline sites and a meta-analysis of ring-width series from 11 sites across the Northern Hemisphere (344 shrubs, 575 trees). This paper suggests that under warmer spring conditions of +1°C, a maximum difference exists of 12 days between tree growth beginning and shrubs. Because of winter warming, the process of shrub chilling accumulation and cambial reactivation is delayed. The gap between when separate species begin their growing season in the spring is referred to as phenological escape and is crucial for temperate tree species survival [8]. The authors draw on this mismatch, concluding that moving forward, trees may have a competitive advantage over shrubs for alpine treeline establishment.

I applaud the authors’ creativity and relevance, while offering two main points to consider from western North American research, suggesting some regional variation in tree-shrub dynamics at cold mountain treelines. First, heat-induced drought stress impacted the highest treeline elevational extent along the spine of the Rocky Mountains, where seedling occurrence is now mostly confined to north-facing slopes [9] and nonexistent since 2010 [10]. Consequently, outbreaks of native spruce beetle (*Dendroctonus rufipennis* Kirby) have proven lethal for all tree size classes, extending up into the willow (*Salix* spp) shrub belt (Fig. 1) [9]. Drought stress reinforces how essential phenological escape is, so seedlings can assimilate more carbon in spring, compensating for higher respiration costs later in the growing season [8]. Second, willow shrubs expanded by 441% across alpine tundra since 1946 in the Colorado Front Range, with 78% from clonal growth and only 22% from seed [11]. These results raise questions as to how disturbance and reproductive strategies could influence regional tree-shrub dynamics. Yet, overall, given the salience of this topic for better understanding high-elevation mountain environments in the wake of ongoing climate change, this research provides a strong natural springboard for future research examining tree-shrub dynamics.

**Figure 1.**
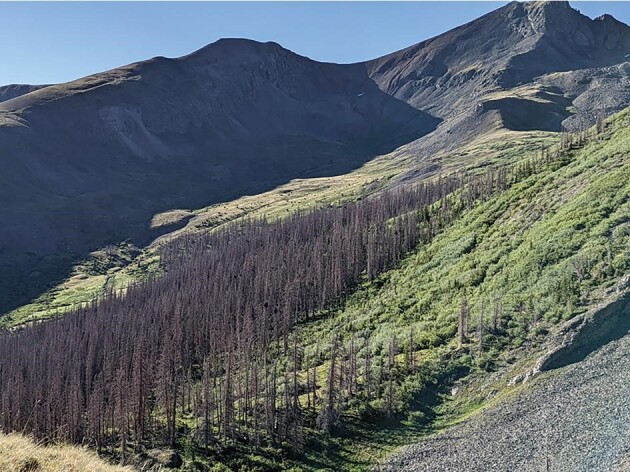
Example of disturbance from spruce beetle-induced mortality affecting tree-shrub dynamics at alpine treeline in the Southern Rocky Mountains, USA. Photo from July 2022 and photo credit to Corey Aldred.

## References

[1] McDowell NG , Allen CD, Anderson-Teixeira K et al. Science 2020; 368: eaaz9463. 10.1126/science.aaz9463 32467364

[2] Mountain Research Initiative EDW Working Group . Nat Clim Change 2015; 5: 424–30. 10.1038/nclimate2563

[3] Lu X , Liang E, Wang Y et al. Glob Ecol Biogeogr 2021; 30: 305–15. 10.1111/geb.13214

[4] Myers-Smith IH , Hik DS. J Ecol 2018; 106: 547–60. 10.1111/1365-2745.12817

[5] Kambo D , Danby RK. Ecosphere 2018; 9: e02176. 10.1002/ecs2.2176

[6] Liang E , Wang Y, Piao S et al. Proc Natl Acad Sci USA 2016; 113: 4380–5. 10.1073/pnas.1520582113 27044083 PMC4843427

[7] Li X , Liang E, Camarero JJ et al. Natl Sci Rev 2023; 10: nwad182. 10.1093/nsr/nwad182 37671321 PMC10476895

[8] Lee BR , Ibáñez I. Funct Ecol 2021; 35: 1848–61. 10.1111/1365-2435.13821

[9] Elliott GP , Bailey SN, Cardinal SJ. Ann Am Assoc Geogr 2021; 111: 756–70. 10.1080/24694452.2020.1805292

[10] Bailey SN , Elliott GP, Schliep EM. Ecosphere 2021; 12: e03568. 10.1002/ecs2.3568

[11] Formica A , Farrer EC, Ashton IW et al. Arct Antarct Alp Res 2014; 46: 616–31. 10.1657/1938-4246-46.3.616

